# ASH structure alignment package: Sensitivity and selectivity in domain classification

**DOI:** 10.1186/1471-2105-8-116

**Published:** 2007-04-04

**Authors:** Daron M Standley, Hiroyuki Toh, Haruki Nakamura

**Affiliations:** 1 Institute for Protein Research, Osaka University, 3-2 Yamadaoka, Suita, Osaka 565-0871, Japan; 2 Japan Science and Technology Agency, Institute for Bioinformatics Research and Development (BIRD), Saitama, Japan; 3 Medical Institute of Bioregulation, Kyushu University, 3-1-1, Maidashi, Higashi-ku, Fukuoka, Fukuoka 812-8582, Japan

## Abstract

**Background:**

Structure alignment methods offer the possibility of measuring distant evolutionary relationships between proteins that are not visible by sequence-based analysis. However, the question of how structural differences and similarities ought to be quantified in this regard remains open. In this study we construct a training set of sequence-unique CATH and SCOP domains, from which we develop a scoring function that can reliably identify domains with the same CATH topology and SCOP fold classification. The score is implemented in the ASH structure alignment package, for which the source code and a web service are freely available from the PDBj website .

**Results:**

The new ASH score shows increased selectivity and sensitivity compared with values reported for several popular programs using the same test set of 4,298,905 structure pairs, yielding an area of .96 under the receiver operating characteristic (ROC) curve. In addition, weak sequence homologies between similar domains are revealed that could not be detected by BLAST sequence alignment. Also, a subset of domain pairs is identified that exhibit high similarity, even though their CATH and SCOP classification differs. Finally, we show that the ranking of alignment programs based solely on geometric measures depends on the choice of the quality measure.

**Conclusion:**

ASH shows high selectivity and sensitivity with regard to domain classification, an important step in defining distantly related protein sequence families. Moreover, the CPU cost per alignment is competitive with the fastest programs, making ASH a practical option for large-scale structure classification studies.

## Background

The last decade has witnessed enormous growth in our knowledge of gene sequences. Efforts are now being made to put this knowledge into a structural context by determining the structures of proteins associated with all known gene families. Protein structure alignment methods are essential for interpreting such data, as they provide a means for detecting evolutionary and functional relationships between distantly related proteins[1]. In practice, however, the problem of quantifying evolutionary distance beyond what is observable through sequence analysis is far from simple. In particular, it is not clear what measure should be used to compare structural domains, and what threshold should be used to judge if they are likely to be related.

These questions were investigated in two recent studies by Sierk and Pearson[2] and by Kolody, *et al.*[3], where a number of structure alignment methods were tested in terms of their ability to correctly identify domains with the same CATH[4] topology. The sensitivity and selectivity of each structure alignment method was assessed in terms of the ratio of true positives (domains with the same CATH topology scoring above a certain threshold) to false positives (domains with different CATH topology scoring above the same threshold). Plotting the true positive ratio against the false positive ratio yields the receiver operating characteristic (ROC) curve, the area under which can be interpreted as "the probability of making a correct choice" given two observations, one true and one false[5]. In the context of domain classification, an "observation" corresponds to a pair of structures. In this case, complications arise that make a definitive comparison of methods difficult.

A major problem concerns the distinction between "true" and "false" (i.e., belonging to the same fold or topology). In any given domain classification system there are borderline cases where a high alignment score is not actually "wrong", even though the two domains may be classified as having different topologies. Conversely, domains classified as belonging to the same topology do not always have optimal alignment scores. In the present work, we modified the ROC method in order to reduce the noise introduced by a binary classification scheme. Specifically, we constructed a new training set of domains and used two domain classifiers, CATH and SCOP[6], for each domain. Given this new training set, and a more "fuzzy" definition of truth, we then derived a general score that showed increased selectivity as a function of sensitivity when compared with other methods, even when applied to a different test set[3] using CATH as the gold standard.

## Implementation

### Derivation of a new training set

The training set used in the present work was constructed using both CATH and SCOP domain definitions. In the first step, the sequence boundaries for each domain from CATH version 3.0.0 and SCOP version 1.69 with a common PDB ID were compared; the domains were considered equivalent if 75% or more of the residues of the larger domain were shared. A total of 63,010 domains were compared in this way, resulting in 43,773 equivalent domains, for which the CATH domains boundaries were then used. A representative subset of the equivalent domains was then derived using the following procedure:

1. A BLAST alignment was computed for each pair of domain sequences.

2. The sequences were combined using single-linkage clustering with an e-value cutoff of 0.1 to produce an initial set of sequence families.

3. Each initial sequence family was then partitioned by the following iterative procedure:

a. The member with the greatest number of links was chosen, and a new cluster defined with it as the representative and all of its links members.

b. The representative and all its members were removed and step 3a is repeated until there were no members left.

The representatives defined in 3a (above) define the non-redundant sequence set (BLAST e-value > .1) which yielded a total of 2911 sequence unique domains (see Additional file 1).

A reduced training set was prepared that contained no significant sequence homologs to the test sets (described below). The reduced training set was prepared by comparing each sequence in the full training set to each sequence in the test set using BLAST; if the sequence identity was higher than 30% and the BLAST E-value was lower than 0.001, the domain was removed from the training set. A total of 2514 domains were removed in this way, resulting in a reduced training set of 1397 domains. The ASH program is released with parameters derived from the full training set. In the following sections, we refer to parameter values optimized on both the full and on the reduced training set.

### Modified ROC curves

The ROC curves computed for the training set treat the CATH and SCOP classifications as independent measures of "truth". Consider a given query-template pair scoring above a given score threshold. If both measures classify the pair as belonging to the same topology/fold, the true-positive value was increased by 2; if only one of the measures classifies them as "true", both the true-positive and false-positive values were increased by 1; and, if they both classify the pair is "false" the false-positive value was increased by 2. When reporting the ratio of false positives and true positives, the values were normalized by their sum over the entire training set, so the resulting curve had the dimensions of a normal ROC curve.

All evaluations performed on the test set used the traditional ROC curve procedure, with only CATH topology classifications. This enabled direct comparison with the performance reported by Koldny, *et al. *[3].

### Derivation of an improved scoring function

In the new ASH scoring function, we combined two measures of "similarity" (a structure term and a sequence term) with three measures of "error" (an RMSD-based term, a gap penalty, and an alignment-independent term):

*Score *= *W*_*NER*_*NER*^*rel *^+ *W*_*seq*_*Fseq *- *W*_*SAS*_*SAS *- *W*_*gap*_*Gap *- *W*_*Dist*_*Dist*.

In this section we will describe each term as it appears in equation 1. The weights are parameters that were used to maximize the area under a ROC curve for the training set described above.

The number of equivalent residues (NER) function was introduced previously[7]. Here we used the normalized form, which is a sum over all *N *aligned residue pairs of a similarity function *S*:

NERSrel=∑iNSi12(Nq+Nt),
 MathType@MTEF@5@5@+=feaafiart1ev1aaatCvAUfKttLearuWrP9MDH5MBPbIqV92AaeXatLxBI9gBaebbnrfifHhDYfgasaacH8akY=wiFfYdH8Gipec8Eeeu0xXdbba9frFj0=OqFfea0dXdd9vqai=hGuQ8kuc9pgc9s8qqaq=dirpe0xb9q8qiLsFr0=vr0=vr0dc8meaabaqaciaacaGaaeqabaqabeGadaaakeaacqWGobGtcqWGfbqrcqWGsbGudaqhaaWcbaGaem4uamfabaGaemOCaiNaemyzauMaemiBaWgaaOGaeyypa0ZaaSaaaeaadaaeWbqaaiabdofatnaaBaaaleaacqWGPbqAaeqaaaqaaiabdMgaPbqaaiabd6eaobqdcqGHris5aaGcbaWaaSaaaeaacqaIXaqmaeaacqaIYaGmaaWaaeWaaeaacqWGobGtdaWgaaWcbaGaemyCaehabeaakiabgUcaRiabd6eaonaaBaaaleaacqWG0baDaeqaaaGccaGLOaGaayzkaaaaaiabcYcaSaaa@48F4@

where *N*_*q *_and *N*_*t *_are the number residues in the query and template, respectively. The similarity term is a Gaussian function

Sid0=e−(did0)2,
 MathType@MTEF@5@5@+=feaafiart1ev1aaatCvAUfKttLearuWrP9MDH5MBPbIqV92AaeXatLxBI9gBaebbnrfifHhDYfgasaacH8akY=wiFfYdH8Gipec8Eeeu0xXdbba9frFj0=OqFfea0dXdd9vqai=hGuQ8kuc9pgc9s8qqaq=dirpe0xb9q8qiLsFr0=vr0=vr0dc8meaabaqaciaacaGaaeqabaqabeGadaaakeaacqWGtbWudaqhaaWcbaGaemyAaKgabaGaemizaq2aaSbaaWqaaiabicdaWaqabaaaaOGaeyypa0Jaemyzau2aaWbaaSqabeaacqGHsisldaqadaqaamaalaaabaGaemizaq2aaSbaaWqaaiabdMgaPbqabaaaleaacqWGKbazdaWgaaadbaGaeGimaadabeaaaaaaliaawIcacaGLPaaadaahaaadbeqaaiabikdaYaaaaaGccqGGSaalaaa@3E4A@

where *d*_*i *_refers to the distance between two aligned Cα atoms, and the width of the Gaussian, *d*_0_, is a parameter with a default value of 4 Å (used in all data presented here). The optimized weight of the NER term *W*_*NER *_was 0.12 for the full training set and 3.0 for the reduced training set.

The sequence similarity function is the sum of the product of equation 3 and an element of an amino acid exchange matrix *A*. If *k*_*i*_, *l*_*i *_represents the sorted (*k*_*i *_<= *l*_*i*_) pair of amino acids associated with aligned pair *i*, then

Fseq=∑iNSi∗A[ki,li]∑iNSi.
 MathType@MTEF@5@5@+=feaafiart1ev1aaatCvAUfKttLearuWrP9MDH5MBPbIqV92AaeXatLxBI9gBaebbnrfifHhDYfgasaacH8akY=wiFfYdH8Gipec8Eeeu0xXdbba9frFj0=OqFfea0dXdd9vqai=hGuQ8kuc9pgc9s8qqaq=dirpe0xb9q8qiLsFr0=vr0=vr0dc8meaabaqaciaacaGaaeqabaqabeGadaaakeaacqWGgbGrdaWgaaWcbaGaem4CamNaemyzauMaemyCaehabeaakiabg2da9maalaaabaWaaabCaeaacqWGtbWudaWgaaWcbaGaemyAaKgabeaakiabgEHiQiabdgeabnaadmaabaGaem4AaS2aaSbaaSqaaiabdMgaPbqabaGccqGGSaalcqWGSbaBdaWgaaWcbaGaemyAaKgabeaaaOGaay5waiaaw2faaaWcbaGaemyAaKgabaGaemOta4eaniabggHiLdaakeaadaaeWbqaaiabdofatnaaBaaaleaacqWGPbqAaeqaaaqaaiabdMgaPbqaaiabd6eaobqdcqGHris5aaaakiabc6caUaaa@4DCF@

The derivation of the matrix elements of *A *are discussed below. The optimized weight of the sequence term *W*_*seq *_is 1.0 for the full training set and 0.14 for the reduced training set.

The SAS function is proportional to the ratio of the RMSD to the number of aligned residues[8]:

SAS=100N∑iNdi2N.
 MathType@MTEF@5@5@+=feaafiart1ev1aaatCvAUfKttLearuWrP9MDH5MBPbIqV92AaeXatLxBI9gBaebbnrfifHhDYfgasaacH8akY=wiFfYdH8Gipec8Eeeu0xXdbba9frFj0=OqFfea0dXdd9vqai=hGuQ8kuc9pgc9s8qqaq=dirpe0xb9q8qiLsFr0=vr0=vr0dc8meaabaqaciaacaGaaeqabaqabeGadaaakeaacqWGtbWucqWGbbqqcqWGtbWucqGH9aqpdaWcaaqaaiabigdaXiabicdaWiabicdaWaqaaiabd6eaobaadaGcaaqaamaalaaabaWaaabCaeaacqWGKbazdaqhaaWcbaGaemyAaKgabaGaeGOmaidaaaqaaiabdMgaPbqaaiabd6eaobqdcqGHris5aaGcbaGaemOta4eaaaWcbeaakiabc6caUaaa@3FE6@

The optimized weight of the SAS term *W*_*SAS *_was 0.85 for the full training set and 10.9 for the reduced training set.

The Gap term is just the number of "internal" gaps. In order to define "internal" we must first define the alignment boundaries. The beginning (ending) of the alignment was defined to be the first (last) residue for which the average similarity score over the following (previous) 5 residues was greater than 0.55. The optimized weight of the gap term *W*_*gap *_was 0.01 for the full training set and -0.1 for the reduced training set.

The *Dist *term consists of "structural descriptors" that do not depend on the alignment. They are combined in a weighted distance:

Dist=|WNrΔNres2+WRgΔRg2+WcoΔCo2+WαΔNα2+WβΔNβ2WNr2+WRg2+Wco2+Wα2+Wβ2|,
 MathType@MTEF@5@5@+=feaafiart1ev1aaatCvAUfKttLearuWrP9MDH5MBPbIqV92AaeXatLxBI9gBamXvP5wqSXMqHnxAJn0BKvguHDwzZbqegyvzYrwyUfgarqqtubsr4rNCHbGeaGqiA8vkIkVAFgIELiFeLkFeLk=iY=Hhbbf9v8qqaqFr0xc9pk0xbba9q8WqFfeaY=biLkVcLq=JHqVepeea0=as0db9vqpepesP0xe9Fve9Fve9GapdbaqaaeGacaGaaiaabeqaamqadiabaaGcbaGaemiraqKaemyAaKMaem4CamNaemiDaqNaeyypa0ZaaOaaaeaadaWcaaqaaiabdEfaxnaaBaaaleaacqWGobGtcqWGYbGCaeqaaOGaeuiLdqKaemOta4KaemOCaiNaemyzauMaem4Cam3aaWbaaSqabeaacqaIYaGmaaGccqGHRaWkcqWGxbWvdaWgaaWcbaGaemOuaiLaem4zaCgabeaakiabfs5aejabdkfasjabdEgaNnaaCaaaleqabaGaeGOmaidaaOGaey4kaSIaem4vaC1aaSbaaSqaaiabdogaJjabd+gaVbqabaGccqqHuoarcqWGdbWqcqWGVbWBdaahaaWcbeqaaiabikdaYaaakiabgUcaRiabdEfaxnaaBaaaleaaiiGacqWFXoqyaeqaaOGaeuiLdqKaemOta4Kae8xSde2aaWbaaSqabeaacqaIYaGmaaGccqGHRaWkcqWGxbWvdaWgaaWcbaGae8NSdigabeaakiabfs5aejabd6eaojab=j7aInaaCaaaleqabaGaeGOmaidaaaGcbaGaem4vaC1aa0baaSqaaiabb6eaojabbkhaYbqaaiabbccaGiabbccaGiabbkdaYaaakiabgUcaRiabdEfaxnaaDaaaleaacqqGsbGucqqGNbWzaeaacqqGGaaicqqGGaaicqqGYaGmaaGccqGHRaWkcqWGxbWvdaqhaaWcbaGaee4yamMaee4Ba8gabaGaeeiiaaIaeeiiaaIaeeOmaidaaOGaey4kaSIaem4vaC1aa0baaSqaaiabl2==UbqaaiabbccaGiabbkdaYaaakiabgUcaRiabdEfaxnaaDaaaleaacqWI9=VBaeaacqqGGaaicqqGYaGmaaaaaaqabaGccqGGSaalaaa@99C0@

where the individual descriptors are defined in Table 1. The optimized weight of the *Dist *term *W*_*Dist *_was 1.1 for the full training set and 7.12 for the reduced training set.

**Table 1:** Structural descriptors

Term	Definition	Description	Weight	Full TS	Red TS
Δ*Nres*	Nq−Nt(Nq+Nt)/2 MathType@MTEF@5@5@+=feaafiart1ev1aaatCvAUfKttLearuWrP9MDH5MBPbIqV92AaeXatLxBI9gBaebbnrfifHhDYfgasaacH8akY=wiFfYdH8Gipec8Eeeu0xXdbba9frFj0=OqFfea0dXdd9vqai=hGuQ8kuc9pgc9s8qqaq=dirpe0xb9q8qiLsFr0=vr0=vr0dc8meaabaqaciaacaGaaeqabaqabeGadaaakeaadaWcaaqaaiabd6eaonaaBaaaleaacqWGXbqCaeqaaOGaeyOeI0IaemOta40aaSbaaSqaaiabdsha0bqabaaakeaacqGGOaakcqWGobGtdaWgaaWcbaGaemyCaehabeaakiabgUcaRiabd6eaonaaBaaaleaacqWG0baDaeqaaOGaeiykaKIaei4la8IaeGOmaidaaaaa@3D39@	Relative difference in the sequence lengths N_q _and N_t _of query and template, respectively	*W*_*Nr*_	51.94	5.92
Δ*Rg*	*Rg*_*q *_- *Rg*_*t*_	Difference in the radii of gyration of the query and template	*W*_*Rg*_	-0.33	-0.54
Δ*Co*	CoqαNq−CotαNt MathType@MTEF@5@5@+=feaafiart1ev1aaatCvAUfKttLearuWrP9MDH5MBPbIqV92AaeXatLxBI9gBaebbnrfifHhDYfgasaacH8akY=wiFfYdH8Gipec8Eeeu0xXdbba9frFj0=OqFfea0dXdd9vqai=hGuQ8kuc9pgc9s8qqaq=dirpe0xb9q8qiLsFr0=vr0=vr0dc8meaabaqaciaacaGaaeqabaqabeGadaaakeaadaWcaaqaaiabdoeadjabd+gaVnaaDaaaleaacqWGXbqCaeaaiiGacqWFXoqyaaaakeaacqWGobGtdaWgaaWcbaGaemyCaehabeaaaaGccqGHsisldaWcaaqaaiabdoeadjabd+gaVnaaDaaaleaacqWG0baDaeaacqWFXoqyaaaakeaacqWGobGtdaWgaaWcbaGaemiDaqhabeaaaaaaaa@3EB7@	Difference in the relative contact orders of query and template, with the modification that only Cα atoms are used, and the cutoff distance was set to 10 Å	*W*_*co*_	0.96	0.42
Δ*N*α	NqαNq−NtαNt MathType@MTEF@5@5@+=feaafiart1ev1aaatCvAUfKttLearuWrP9MDH5MBPbIqV92AaeXatLxBI9gBaebbnrfifHhDYfgasaacH8akY=wiFfYdH8Gipec8Eeeu0xXdbba9frFj0=OqFfea0dXdd9vqai=hGuQ8kuc9pgc9s8qqaq=dirpe0xb9q8qiLsFr0=vr0=vr0dc8meaabaqaciaacaGaaeqabaqabeGadaaakeaadaWcaaqaaiabd6eaonaaDaaaleaacqWGXbqCaeaaiiGacqWFXoqyaaaakeaacqWGobGtdaWgaaWcbaGaemyCaehabeaaaaGccqGHsisldaWcaaqaaiabd6eaonaaDaaaleaacqWG0baDaeaacqWFXoqyaaaakeaacqWGobGtdaWgaaWcbaGaemiDaqhabeaaaaaaaa@3C15@	Differences in the relative number of helical residues	*W*_α_	3.21	0.68
Δ*N*β	NqβNq−NtβNt MathType@MTEF@5@5@+=feaafiart1ev1aaatCvAUfKttLearuWrP9MDH5MBPbIqV92AaeXatLxBI9gBaebbnrfifHhDYfgasaacH8akY=wiFfYdH8Gipec8Eeeu0xXdbba9frFj0=OqFfea0dXdd9vqai=hGuQ8kuc9pgc9s8qqaq=dirpe0xb9q8qiLsFr0=vr0=vr0dc8meaabaqaciaacaGaaeqabaqabeGadaaakeaadaWcaaqaaiabd6eaonaaDaaaleaacqWGXbqCaeaaiiGacqWFYoGyaaaakeaacqWGobGtdaWgaaWcbaGaemyCaehabeaaaaGccqGHsisldaWcaaqaaiabd6eaonaaDaaaleaacqWG0baDaeaacqWFYoGyaaaakeaacqWGobGtdaWgaaWcbaGaemiDaqhabeaaaaaaaa@3C19@	Differences in the relative number of strand residues	*W*_β_	1.71	0.76

The structural descriptor terms used in the ASH score are listed. Each descriptor is independent of alignment. The optimized weights of each term for the full training set (Full TS) and reduced training set (Red TS) are listed in the last two columns.

### Amino acid substitution matrix

The amino acid substitution matrix was derived from statistics drawn from the training set. The elements of *A *are defined traditionally, as the log of the observed frequency of aligned residue pairs over the expected frequency:

A[x,y]=log⁡2(pxyobsPxyexp⁡)
 MathType@MTEF@5@5@+=feaafiart1ev1aaatCvAUfKttLearuWrP9MDH5MBPbIqV92AaeXatLxBI9gBaebbnrfifHhDYfgasaacH8akY=wiFfYdH8Gipec8Eeeu0xXdbba9frFj0=OqFfea0dXdd9vqai=hGuQ8kuc9pgc9s8qqaq=dirpe0xb9q8qiLsFr0=vr0=vr0dc8meaabaqaciaacaGaaeqabaqabeGadaaakeaacqWGbbqqcqGGBbWwcqWG4baEcqGGSaalcqWG5bqEcqGGDbqxcqGH9aqpcyGGSbaBcqGGVbWBcqGGNbWzdaWgaaWcbaGaeGOmaidabeaakmaabmaabaWaaSaaaeaacqWGWbaCdaqhaaWcbaGaemiEaGNaemyEaKhabaGaem4Ba8MaemOyaiMaem4CamhaaaGcbaGaemiuaa1aa0baaSqaaiabdIha4jabdMha5bqaaiGbcwgaLjabcIha4jabcchaWbaaaaaakiaawIcacaGLPaaaaaa@4D2F@

where *x*, *y *represents an arbitrary sorted (*x *<= *y*) pair of amino acid types. We utilized equation 3 in defining the frequency of each substitution:

px,yobs=∑jM∑iNj{Sijif(ki=xandli=y)0otherwise∑jM∑iNjSij
 MathType@MTEF@5@5@+=feaafiart1ev1aaatCvAUfKttLearuWrP9MDH5MBPbIqV92AaeXatLxBI9gBaebbnrfifHhDYfgasaacH8akY=wiFfYdH8Gipec8Eeeu0xXdbba9frFj0=OqFfea0dXdd9vqai=hGuQ8kuc9pgc9s8qqaq=dirpe0xb9q8qiLsFr0=vr0=vr0dc8meaabaqaciaacaGaaeqabaqabeGadaaakeaacqWGWbaCdaqhaaWcbaGaemiEaGNaeiilaWIaemyEaKhabaGaem4Ba8MaemOyaiMaem4CamhaaOGaeyypa0ZaaSaaaeaadaaeWbqaamaaqahabaWaaiqaaeaafaqaaeGabaaabaqbaeqabeGaaaqaaiabdofatnaaDaaaleaacqWGPbqAaeaacqWGQbGAaaaakeaafaqabeqadaaabaGaemyAaKMaemOzayMaeiikaGIaem4AaS2aaSbaaSqaaiabdMgaPbqabaGccqGH9aqpcqWG4baEaeaacqWGHbqycqWGUbGBcqWGKbazaeaacqWGSbaBdaWgaaWcbaGaemyAaKgabeaakiabg2da9iabdMha5baaaaGaeiykaKcabaqbaeqabeGaaaqaaiabicdaWaqaaiabd+gaVjabdsha0jabdIgaOjabdwgaLjabdkhaYjabdEha3jabdMgaPjabdohaZjabdwgaLbaaaaaacaGL7baaaSqaaiabdMgaPbqaaiabd6eaonaaCaaameqabaGaemOAaOgaaaqdcqGHris5aaWcbaGaemOAaOgabaGaemyta0eaniabggHiLdaakeaadaaeWbqaamaaqahabaGaem4uam1aa0baaSqaaiabdMgaPbqaaiabdQgaQbaaaeaacqWGPbqAaeaacqWGobGtdaahaaadbeqaaiabdQgaQbaaa0GaeyyeIuoaaSqaaiabdQgaQbqaaiabd2eanbqdcqGHris5aaaaaaa@77AF@

where *k*_*i*_*l*_*i *_represents the (sorted) pair of amino acids associated with aligned pair *i*. Equation 8 essentially means that each amino acid substitution is weighted by equation 3, which is a function of the Euclidian distance between the two residues in the ASH superposition. The expected frequency is just the product of the individual amino acid frequencies

px,yexp⁡=pxobs∗pyobs
 MathType@MTEF@5@5@+=feaafiart1ev1aaatCvAUfKttLearuWrP9MDH5MBPbIqV92AaeXatLxBI9gBaebbnrfifHhDYfgasaacH8akY=wiFfYdH8Gipec8Eeeu0xXdbba9frFj0=OqFfea0dXdd9vqai=hGuQ8kuc9pgc9s8qqaq=dirpe0xb9q8qiLsFr0=vr0=vr0dc8meaabaqaciaacaGaaeqabaqabeGadaaakeaacqWGWbaCdaqhaaWcbaGaemiEaGNaeiilaWIaemyEaKhabaGagiyzauMaeiiEaGNaeiiCaahaaOGaeyypa0JaemiCaa3aa0baaSqaaiabdIha4bqaaiabd+gaVjabdkgaIjabdohaZbaakiabgEHiQiabdchaWnaaDaaaleaacqWG5bqEaeaacqWGVbWBcqWGIbGycqWGZbWCaaaaaa@46B9@

where

pxobs=∑jM∑iNj{Sijif(ki=xorli=x)0otherwise∑jM∑iNjSij.
 MathType@MTEF@5@5@+=feaafiart1ev1aaatCvAUfKttLearuWrP9MDH5MBPbIqV92AaeXatLxBI9gBaebbnrfifHhDYfgasaacH8akY=wiFfYdH8Gipec8Eeeu0xXdbba9frFj0=OqFfea0dXdd9vqai=hGuQ8kuc9pgc9s8qqaq=dirpe0xb9q8qiLsFr0=vr0=vr0dc8meaabaqaciaacaGaaeqabaqabeGadaaakeaacqWGWbaCdaqhaaWcbaGaemiEaGhabaGaem4Ba8MaemOyaiMaem4CamhaaOGaeyypa0ZaaSaaaeaadaaeWbqaamaaqahabaWaaiqaaeaafaqaaeGabaaabaqbaeqabeGaaaqaaiabdofatnaaDaaaleaacqWGPbqAaeaacqWGQbGAaaaakeaafaqabeqadaaabaGaemyAaKMaemOzayMaeiikaGIaem4AaS2aaSbaaSqaaiabdMgaPbqabaGccqGH9aqpcqWG4baEaeaacqWGVbWBcqWGYbGCaeaacqWGSbaBdaWgaaWcbaGaemyAaKgabeaakiabg2da9iabdIha4baacqGGPaqkaaaabaqbaeqabeGaaaqaaiabicdaWaqaaiabd+gaVjabdsha0jabdIgaOjabdwgaLjabdkhaYjabdEha3jabdMgaPjabdohaZjabdwgaLbaaaaaacaGL7baaaSqaaiabdMgaPbqaaiabd6eaonaaCaaameqabaGaemOAaOgaaaqdcqGHris5aaWcbaGaemOAaOgabaGaemyta0eaniabggHiLdaakeaadaaeWbqaamaaqahabaGaem4uam1aa0baaSqaaiabdMgaPbqaaiabdQgaQbaaaeaacqWGPbqAaeaacqWGobGtdaahaaadbeqaaiabdQgaQbaaa0GaeyyeIuoaaSqaaiabdQgaQbqaaiabd2eanbqdcqGHris5aaaakiabc6caUaaa@7513@

In order to avoid over-fitting of the amino acid substitution matrix, cross validation was performed to ensure that the result was not sensitive to the exclusion and any single domain in the training set (a separate calculation was performed for the reduced and full training sets). This involved computing a different substitution matrix for each domain in the training set, wherein the domain in question was excluded. The unbiased matrix was obtained by rounding each term in each matrix until there were no differences between any two matrices. The final amino acid substitution matrix for the full training set is shown in Additional file 2.

### ASH 1.0 structure alignment package

In an earlier study we described a web service (GASH) that utilizes the double dynamic programming algorithm in conjunction with maximization of the NER score (equation 2) using a quasi-genetic algorithm[9]. In the current work, we present a streamlined version of the methodology that is released both as a web service and as a suite of command-line programs, including: a faster version of GASH; a streamlined alignment program (crossover step omitted) for more rapid pair-wise alignment (RASH); a batch version of RASH for processing a list of templates (LASH), and a utility program for converting PDB-formatted files to the native data structure used by ASH (CONVERT). The programs are written in ANSI C, and the source code is freely available from the ASH webserver[10].

Several improvements have been made to reduce the computational time of all the above programs:

1. More efficient integration between Local ASH and the NER maximization procedures.

2. Optimization of the double dynamic programming routine. In particular, attention was spent on the lower dynamic programming (DP) step, since, in principle, this step must be repeated for every aligned residue pair. We now only invoke the lower DP if the residue pairs in question have the same secondary structure assignment.

3. The "local environment" of each residue is limited to a sequence window of +/- 10 residues.

4. The default behavior in RASH and LASH is to skip the crossover step used in GASH.

5. When processing a list of templates using LASH you must pre-compute the local environments and store these as text files. This reduces the overhead of repeatedly computing the local environments internally.

Together, these improvements reduce the time per alignment for LASH by approximately a factor of 20 relative to the original GASH program.

### Reference alignment programs

Four external structure alignment programs were used directly in this study: DaliLite[11] was downloaded from the FSSP server[12]; STRUCTAL[13] was taken from the STRUCTAL download page[14]; FAST[15] was taken from the FAST server[16]; SSM [17] was taken from the SSM download center[18].

### Test sets

The test set was taken from Kolodny, *et al. *and consisted of all unique, non-equal pairs of 2,930 sequence-unique CATH domains [3]. A subset of this set was used for geometric analysis and benchmarking. For geometric assessment we consider the "challenging" alignment pairs identified by Kolodny, *et al. *for which the CATH topology is identical. The resulting test set of structure pairs is significantly smaller (2,070) than that used for the domain classification test. However, the query-template pairs have been selected from the full test set based on the fact that only one of the alignment methods tested in Kolodny, *et al. *produced a "good" alignment [3]. Thus we expect that any systematic differences in the methods will be amplified in our comparison.

## Results and Discussion

### ROC analysis

**Figure 1:**
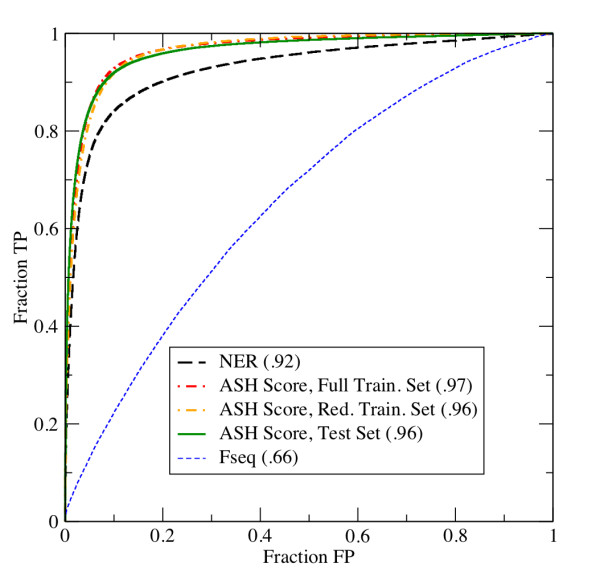
**ROC Curves**. The ROC curve for the NER score (black), the new ASH score, and the sequence similarity term (blue) are shown. The new ASH score was evaluated both on the full training set (red), on the reduced training set (orange), and the test set using parameters derived from the reduced training set (green). The area under each curve is indicated in the legend.

Figure 1 shows the ROC curves for all 2930 × 2929/2 unique, non-identical structure pairs from the test set. All results were obtained using the LASH program with parameters derived from the reduced training set. First we consider the sensitivity and selectivity of the NER score alone. The area under the NER ROC curve is .92, slightly worse than Dali and STRUCTAL, for which the corresponding area was .94[3]. This non-optimal yet encouraging result motivated us to develop the new ASH scoring function.

Using the new training set, we optimized the weights of the new scoring function using a Monte Carlo procedure. Figure 1 shows the ROC curve for the full training set, for which the area under the curve is .97. In order to put this number into perspective, we examined the highest scoring template for each query. Specifically, we evaluated the frequency of making a "false" choice (according to both CATH and SCOP classifications) when only the highest scoring template for a given query was used. There were 69 such mistakes out of 1,641 queries for which a template exists with both the same CATH and SCOP classification (see Additional file 3). This number was small enough that inspection of the alignments by eye was feasible. In general, the "false positive" pairs appeared to be very similar, in many cases more similar than the highest-scoring "true" template. This result assured us that high-scoring alignments make physical sense, even if they are classified as belonging to different topologies or folds.

As Figure 1 shows, the area under the curve drops slightly to .96 when the training is done on the reduced training set. However, the area remains at .96 when applied to the test set, no matter which set of parameters is used, indicating that the parameters have not been over-fit. The difference between ASH and the best reported result is 2% for the test set[3]. While in relative terms this number is quite small, the absolute number of false positives could be significant if a large number of queries were performed.

We also evaluated the ROC curve for the sequence similarity term alone. As Figure 1 shows, this curve indicates a very weak sequence signal among the "true" pairs in the test set. For reference, a random score would be expected to approximate the function y = x. Thus, while the sequence term does not provide a large amount of information in this non-redundant test set, it may help in distinguishing between borderline cases. Moreover, it provides a link between sequence and structure information for the general cases where sequence homologs have not been excluded *a priori*.

The matrix elements of the derived substitution matrix were compared with those of the BLOSUM62 matrix[19] (see Additional file 4). The slope and correlation coefficient for this plot are .52 and .94, respectively. As a reference, we also plot the matrix elements derived from all domain pairs with different CATH and SCOP topology/fold values. The corresponding slope and correlation coefficient are 0.06 and 0.70, respectively. This clearly indicates a sequence relationship between members with the same CATH topology and SCOP fold that is weak, yet stronger than that observed between members with different CATH topology and SCOP fold identifiers.

To further investigate the similarity between the proposed substitution matrix and previous work by other groups, our matrix was compared to all 98 entries in the AAindex, a database of substitution matrices [20-22]. After centering, and normalizing each matrix, we considered two criteria of similarity: the dot-product and the RMSD of the matrix values. The most similar matrix according to the dot-product was that of Gonnet, *et al. *[23] with a value of .89. Interestingly, this matrix was found to be the most accurate of 30 different matrices in an assessment by Vogt, et al. [24]. The most similar according to RMSD was the SDM matrix of Prlic, *et al. *[25], with a value of .13. The SDM matrix was derived by a method similar to ours and found to perform slightly better than that of Gonnet, *et al. *[25]. These results suggest that the proposed amino acid substitution matrix may have utility in sequence based alignment studies, a question we intend to investigate in the future.

Since we have followed the ROC curve procedure used by Kolodny, *et al. *to evaluate our domain classification scheme, it is worthwhile to mention that, in their study, Kolodny, *et al. *state that "the number of disadvantages of using ROC curves methodology for comparing structure alignment methods exceeds the number of potential advantages[3]" They argue, rather, for a direct "geometric" comparison using a well-defined analog function such as the SAS score. We initially were also skeptical about ROC analysis using a single gold standard, such as CATH. Indeed, the drop in area from .97 to .96 when applying the new score to the test set (for which only CATH definitions were used) was initially attributed to inherent limitations in the CATH classification scheme, rather than over-fitting on the training set or some other cause. In order to directly investigate this question, we repeated the calculation on the training set using each of the single classifications (CATH and SCOP) separately. To our surprise, the resulting ROC areas remained at .97, suggesting that binary classification was not a significant problem. In the next section, we investigate the quality of the alignments themselves using geometric measures.

### Geometric analysis

**Figure 2:**
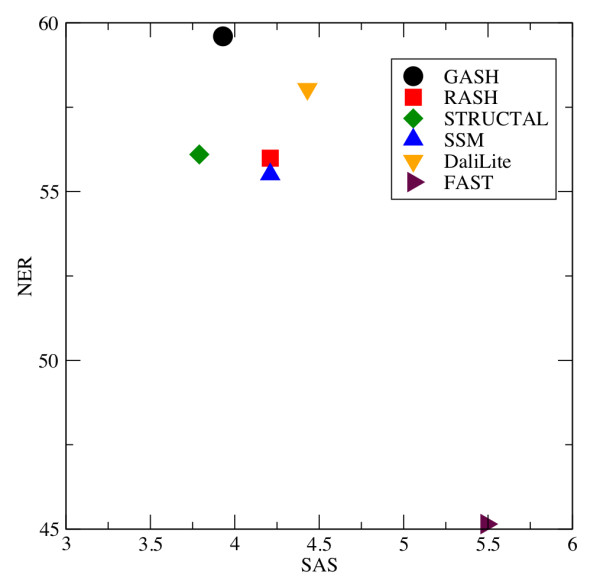
**Geometric Analysis**. The relationship between average NER score and average SAS score. The average was computed over 2,071 query-template pairs using the geometric test set.

In our geometric assessment we compute the average SAS and NER scores derived from 6 alignment programs: GASH, RASH, DaliLite, FAST, SSM, and STRUCTAL. We use the smaller "challenging" test set of true (same CATH topology) matches, as described in Methods. If any program failed to produce an alignment, we excluded the query-template pair from the average for all methods, and we recorded the frequency of such "missed alignments" in Table 2. In Figure 2, we plot the average NER score vs. the average SAS score for the geometric test set. Since the SAS score is an error function, and the NER score is a similarity function, we might expect to see the best program in the upper-left corner and the worst one in the lower-right. While FAST performs relatively poorly with regard to both NER and SAS, and occupies the lower-right corner the other programs are distributed in a cluster in the upper left and it is hard to pick a clear "winner".

**Table 2:** CPU usage for geometric test set.

Program	CPU (s)	CPU/Align	Rel. to FAST	Align Missing
Dali	12895	6.2	20	121
SRUCTAL	3703	1.8	5	0
GASH	3197	1.5	5	4
RASH	1424	.69	2	0
FAST	645	.31	1	3

The total CPU usage for 5 programs was computed over the geometric test set of 2,071 query-template pairs. The last column lists the number of query-template pairs for which no alignment was obtained by the method.

With regard to the SAS score, STRUCTAL does the best, in agreement with the findings of Kolodny, *et al.*[3], followed by GASH; however, when we consider the NER score, GASH performs the best, followed by DaliLite. RASH and SSM perform at an intermediate level in terms of both scores and very similar to each other. The fact that no single program stands out in terms of both NER and SAS suggests that they are somewhat independent measures. Clearly, care must be used in using a single score or a single class of scores (i.e., error functions vs. similarity functions) as a basis for judging alignment quality. The aggregate function used in ASH takes this independence into account by combining both similarity measures and difference measures. Our overall conclusion regarding geometric analysis is that the "best" alignment method depends on the choice of the geometric measure. Moerover, on average, ASH, STRUCTAL, DaliLite, and SSM all appear to be nearly equal.

### CPU usage

The total CPU required to run LASH on all 2930 × 2930 pairs of structures in the test set was 891 hours, or .37 seconds per alignment on an Intel Pentium4 3.2 GHz personal computer (PC). In order to benchmark ASH programs against reference programs, a head-to-head comparison using the 2,071 query-template pairs in the geometric test set was carried out (Table 2). In this test, FAST was the fastest program at .31 seconds per alignment, but at a significant loss in terms of alignment quality, as noted above. RASH was about a factor of 2 slower, at .69 seconds per alignment. GASH and STRUCTAL were each about a factor of 2 slower than RASH, followed by DaliLite. In their assessment, Kolodny, *et al. *found SSM to be the fastest program. We also found SSM to be very fast when a single query was run against a precompiled list of templates. However, for the present exercise we wished to run a particular set of pairs of structures. In this particular mode, the existing SSM software was not designed to use precompiled results, which caused a considerable increase in CPU time (Eugene Krissinel, personal communication). Since this mode did not reflect SSM performance due to purely technical reasons, the CPU times for SSM were excluded from Table 2.

## Conclusion

We have investigated the utility of using the ASH structure alignment package to recognize domains with the same CATH topology and SCOP fold classifiers. Overall, we can argue that such domains share a distant evolutionary relationship. This can be seen in the strong correlation between the amino acid substitution matrix derived from the aligned "positives" and the BLOSUM62 matrix. The low number of false-positives, suggests that the ASH score correlates with evolutionary distance beyond the "twilight zone" where sequence alignment methods fail. A close examination of the "false positives" produced by ASH suggests that there are some domain pairs that perhaps ought to be considered distant relatives, even though they are classified into different topologies/folds by CATH and SCOP. However, the poor correlation between the substitution matrix derived from different topologies/folds and BLOSUM62 suggests that such cases are rare exceptions. Our study also shows that ASH is competitive with the best programs examined in terms of both geometric scoring and CPU usage.

## Availability and requirements

Project name: ASH

Project home page: 

Operating System(s): Unix-like (successfully compiled on Red Hat Linux and Mac OS X)

Programming language: ANSI C

License: Protein Data Bank Japan Software License, based on FreeBSD.

Any restrictions to use by non-academics: None

## Abbreviations

ROC, receiver operating characteristic curve; CPU central processing unit; PDB, Protein Data Bank; PDBj, Protein Data Bank Japan; CPU; RMSD root mean square deviation; E-value, expectation value; NER, number of equivalent residues; ANSI, American National Standards Institute; PC, personal computer.

## Authors' contributions

DS developed the new scoring function, integrated score with local ASH, performed the alignments, and drafted the manuscript. HT wrote local ASH and relevant sections of the manuscript. HN conceived of the study and participated in its design and coordination. All authors participated in preparation of the manuscript and approved the final version.

## Supplementary Material

Additional File 1 Training set. The file contains the CATH IDs for each domain used in the training set. Click here for file

Additional File 2 Amino acid substitution matrix. The file contains the amino acid substitution matrix values derived from the full training set. Click here for file

Additional File 3 False Positives and True Negatives. For each query, the highest-scoring false positive and the highest-scoring true negative from the training set are listed along with their scores. Click here for file

Additional File 4 Amino acid exchange matrix values compared to the BLOSUM62 matrix. The values of the ASH amino acid substitution matrix derived from true pairs (same CATH topology and same SCOP fold) in the training set are plotted against the corresponding BLOSUM62 matrix elements (black). The corresponding matrix element values derived from false pairs (different CATH topology and different SCOP fold) are shown in red. Click here for file
