# Supplementary figures and images for: ASH structure alignment package: Sensitivity and selectivity in domain classification
Source: BMC Bioinformatics. 2007 Apr 4;8:116. doi: 10.1186/1471-2105-8-116 (PMC1955748; doi:10.1186/1471-2105-8-116)

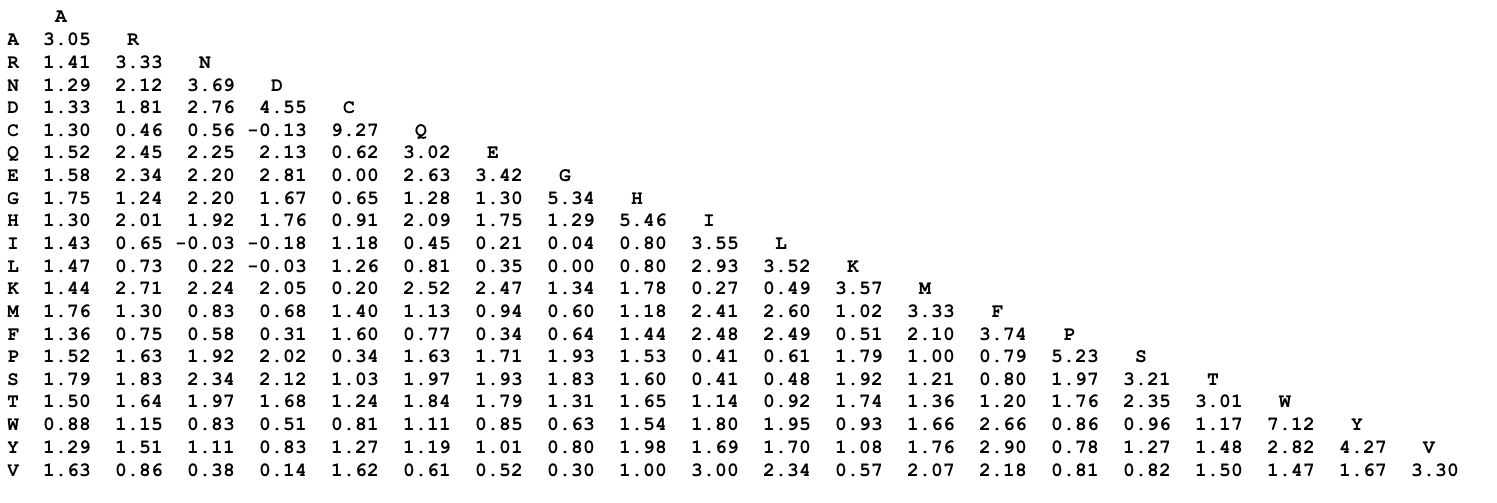

Supplement: Additional File 2 — Amino acid substitution matrix. The file contains the amino acid substitution matrix values derived from the full training set. [file 1471-2105-8-116-S2.tiff]

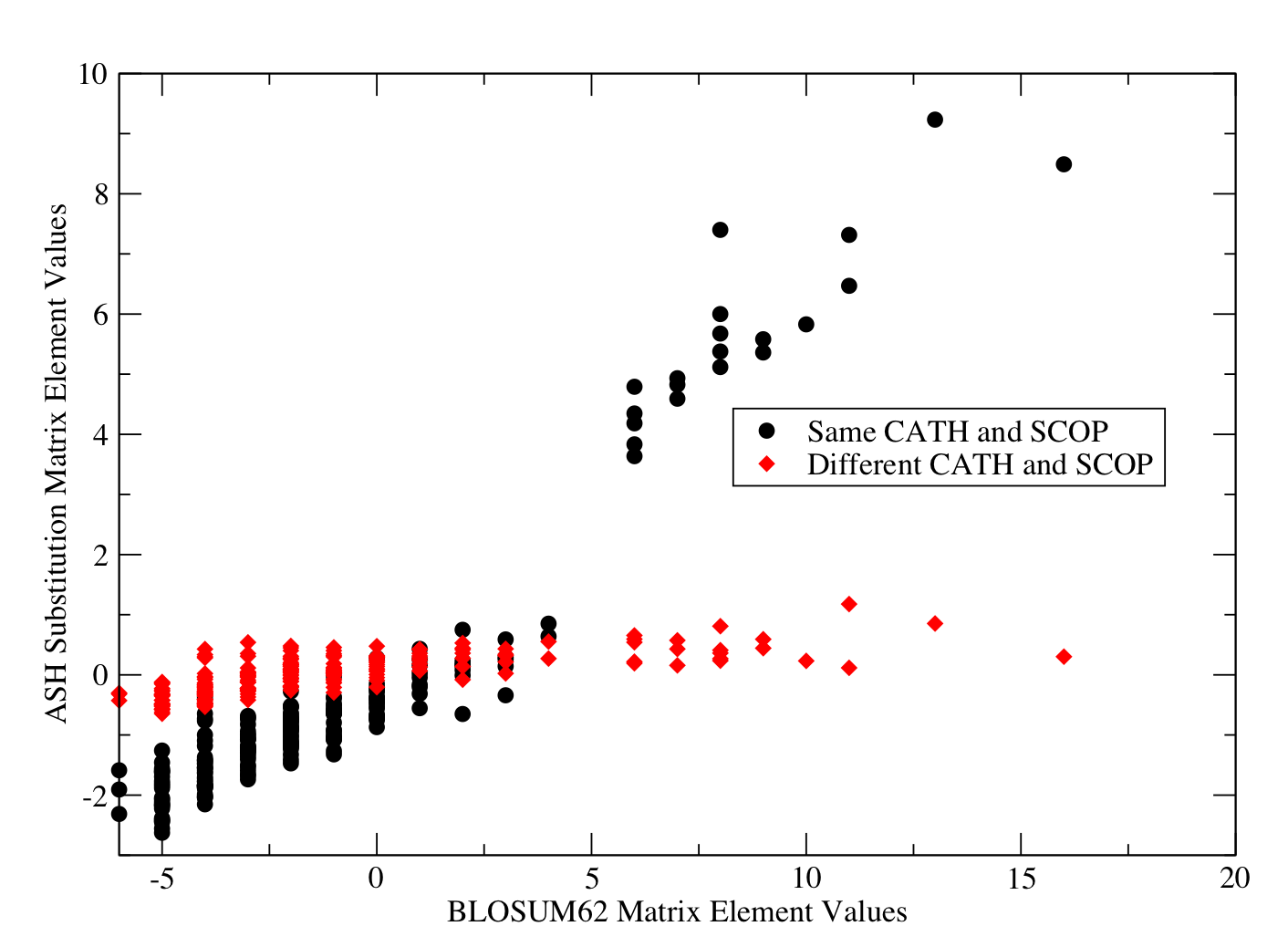

Supplement: Additional File 4 — Amino acid exchange matrix values compared to the BLOSUM62 matrix. The values of the ASH amino acid substitution matrix derived from true pairs (same CATH topology and same SCOP fold) in the training set are plotted against the corresponding BLOSUM62 matrix elements (black). The corresponding matrix element values derived from false pairs (different CATH topology and different SCOP fold) are shown in red. [file 1471-2105-8-116-S4.tiff]
